# Supplementary material for: Incidence of invasive pneumococcal disease after introduction of the 13-valent conjugate pneumococcal vaccine in British Columbia: A retrospective cohort study
Source: PLoS One. 2020 Sep 30;15(9):e0239848. doi: 10.1371/journal.pone.0239848 (PMC7526878; doi:10.1371/journal.pone.0239848)
Supplement: S1 Appendix — (DOCX) [file pone.0239848.s001.docx]

**Supplementary Appendix**

Title: Increase in the incidence of invasive pneumococcal disease from non-pneumococcal conjugate vaccine serotypes in British Columbia: a retrospective cohort study

Running head: Increase in IPD incidence in BC

**Table of Contents Page**

Supplementary eTable1………………………………………………………………………… 2

Supplementary eFigure1………………………………………………………………………. 3

Supplementary eFigure2………………………………………………………………………. 4

Supplementary eFigure3………………………………………………………………………. 5

Supplementary eFigure4……………………………………………………………………… 6

eTable1: Type of invasive pneumococcal disease by age group, British Columbia (Canada), 2002-2015

|  | | |
| --- | --- | --- |
|  | **2002-2015** | |
|  | **Cases** | **%** |
| **Pneumococcal Bacteremia** | | |
| 0-2y | 207 | 5.9 |
| 3-5y | 131 | 3.8 |
| 6-17y | 144 | 4.1 |
| 18-49 | 831 | 23.9 |
| 50-64 | 744 | 21.4 |
| 65-74 | 451 | 12.9 |
| 75-84 | 553 | 15.9 |
| 85+ | 423 | 12.1 |
| **Pneumococcal Septicemia** | | |
| 0-2y | 112 | 5.6 |
| 3-5y | 64 | 3.2 |
| 6-17y | 55 | 2.7 |
| 18-49 | 502 | 25.0 |
| 50-64 | 504 | 25.0 |
| 65-74 | 315 | 15.7 |
| 75-84 | 272 | 13.5 |
| 85+ | 188 | 9.3 |
| **Pneumococcal Meningitis** | | |
| 0-2y | 35 | 11.9 |
| 3-5y | 8 | 2.7 |
| 6-17y | 26 | 8.8 |
| 18-49 | 75 | 25.4 |
| 50-64 | 81 | 27.5 |
| 65-74 | 42 | 14.2 |
| 75-84 | 23 | 7.8 |
| 85+ | 5 | 1.7 |

eFigure1: Seasonal trends of invasive pneumococcal disease, British Columbia (Canada), 2002-2015

eFigure2: Year-on-year analysis of PCV13 serotypes by age group from 2011 to 2015


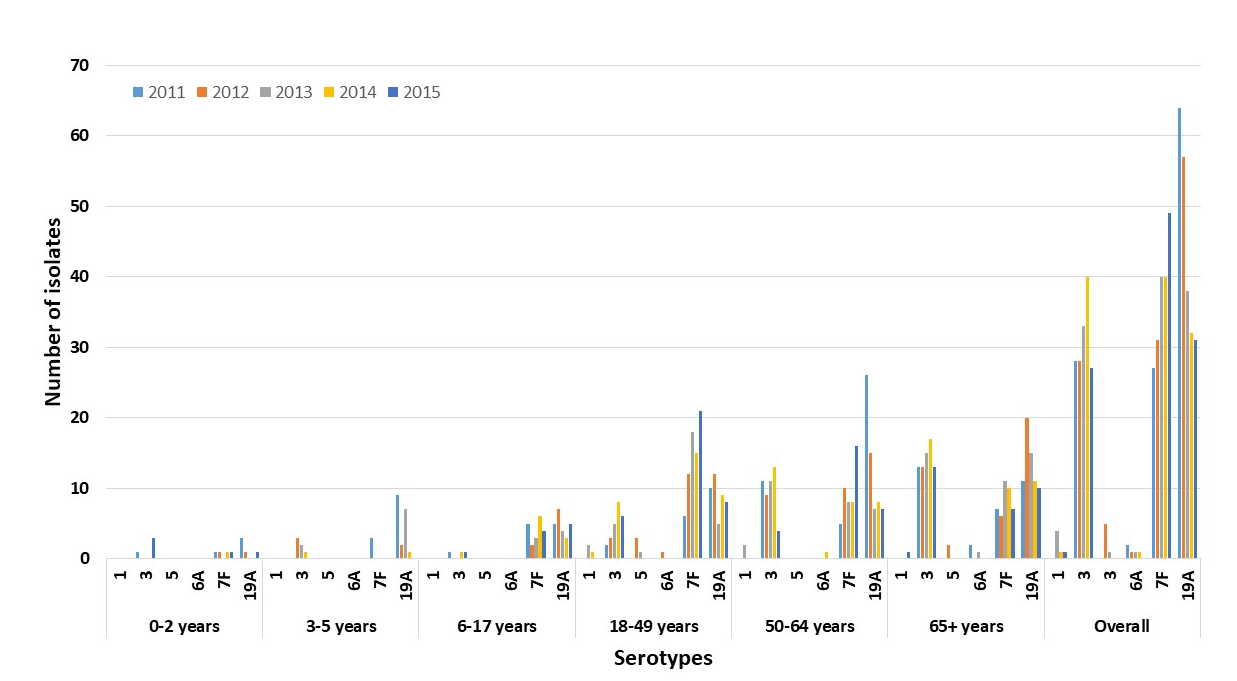


eFigure3: 30-day all-cause mortality in invasive pneumococcal disease patients in British Columbia from 2002-2015 by age group

eFigure4: One year all-cause mortality in invasive pneumococcal disease patients in British Columbia from 2002-2015 by age group
