# Supplementary material for: Incidence of invasive pneumococcal disease after introduction of the 13-valent conjugate pneumococcal vaccine in British Columbia: A retrospective cohort study
Source: PLoS One. 2020 Sep 30;15(9):e0239848. doi: 10.1371/journal.pone.0239848 (PMC7526878; doi:10.1371/journal.pone.0239848)
Supplement: Attachment — Submitted filename: Response to Reviewers_v4.doc [file pone.0239848.s002.doc]

June 17, 2020

Ka Chun Chong

Academic Editor

PLOS ONE

Dear Dr Chong

**RE: Manuscript Number: PONE-D-20-08116**

Thank you for reviewing our paper entitled “Incidence of invasive pneumococcal disease after introduction of the 13-valent conjugate pneumococcal vaccine in British Columbia: a retrospective cohort study” for consideration of publication in the journal of PLOS ONE. We do appreciate the timely feedback received from the reviewers, given the busy period for clinicians right now.

I have endeavored to respond to these comments and have revised the manuscript accordingly. As requested, these changes are in yellow highlights throughout the revised manuscript. Please see my responses to the comments below.

Reviewer #1:

The paper by Vadlamudi et al. describes a retrospective cohort study on the impact of pneumococcal vaccination, either with PCV7 or PCV13, on IPD in British Columbia during 2002-2015. The topic is of importance, but there are some issues that need to be dealt with.

1. The first observation is that the results should be reported in a more comprehensive and organic way, not dividing them in primary and secondary analysis, jumping from the period of transition between PCV7 and PCV13 to the pre vaccination era and vice versa, both for IPD incidence and serotype analysis. It should be more easy for the reader to follow the overall IPD incidence and serotype trends during the entire study period and not going back and forth. As a consequence, Results proved to be quite confusing and should be rewritten avoiding too detailed descriptions of tables.

***Thank you for this suggestion. I have changed the manuscript accordingly.***

2. In addition, Authors should include a briefly description of the case definition and laboratory testing, with the definition of vaccine categories, in Methods and not as supplementary file.

***Thank you for this, I have moved this section from the supplementary file to the methods section. I have deleted the wording “Additional details on the case definition and laboratory testing are presented in the supplementary methods” and revised references accordingly.***

3. For clarity throughout the entire paper, it should be better to report PCV13 serotypes as PCV13-non PCV7 serotypes, and PPV23 serotypes as PPV23-non PCV13 serotypes.

***I would prefer to leave the nomenclature as it currently sits, as “PCV13-non PCV7 serotypes” seems a mouthful and would be confusing the audience. However, in the methods, I have clarified that we have the PCV7 serotypes, then PCV13 serotypes, which refers to “non PCV7 serotypes or delta6”, followed by the PPV23 serotypes, which refers to the additional 11 serotypes, non-vaccine serotypes (not in any PCV or PPV vaccines), and Non-PCV13 serotypes, which are the additional 11 serotypes in PPV23 and NVT.***

4. In Discussion authors should comment more widely the increase observed rates in serotypes 19A and 7F following PCV13 use.

***Thank you. We have added further information regarding these serotypes and current trends in the discussion. We have also augmented our analysis and results in the section on IPD Incidence from the six additional PCV13 serotypes and added a supplemental eFigure 2.***

5. Throughout the entire paper it should be better to denominate the PCV7 and PCV13 periods as PCV7 era and PCV13 era, deleting the prefix ” post”.

***Yes, I agree! I have made this change in the paper, as well in Tables 2 and 3.***

6. Lines 79-80: specify to which years vaccination rate of 80% refers to.

***I have clarified this in lines 80 to 83 as “……the vaccination rate of PCV7 was between 79-83% among the eligible population, between 2007 and 2009[9]. In 2010 BC adopted PCV13, containing an additional six serotypes: 1, 3, 5, 6A, 7F, and 19A for their infant immunization program [10]; the vaccination rate of PCV13 has remained over 82% for the eligible population since 2010[9].***

7. Lines 155-157: description of serotype distribution should be deleted from this paragraph and included in a paragraph dedicated to serotype analysis.

***I have left this as is because it pertains baseline numbers that are in Table 1. Further, the serotype distribution for each vaccine period is presented in specific section of the results and there isn’t a section dedicated to serotypes. Please let me know if I have misunderstood where you meant for this to go, and I would be happy to move it.***

8. It should be useful to include supplementary Figure 2 in the text.

***Thank you. I have moved it into the main text as Figure 3.***

Reviewer #2:

1. This study reviews the results of a provincial-wide surveillance of IPD after the introduction of PCV7 and PCV13 into their children immunization programme during a 14-year period. The paper provides additional information about the effect of pneumococcal conjugate vaccines on IPD incidence and serotype distribution with similar results than those observed elsewhere.

***Thank you for your kind words. We hope the paper will provide valuable information to the readership on invasive pneumococcal disease and our continued evaluation of the immunization programs to stem this disease.***

I do hope this revision meets the journal’s publication standards and requirements. I look forward to hearing back from you soon. Please stay safe!

Sincerely,

Fawziah

**Fawziah Lalji, B.Sc.(Pharm), ACPR, Pharm.D., FCSHP**

Professor | Tong Louie Chair

Faculty of Pharmaceutical Sciences | The University of British Columbia

Vancouver Campus | Musqueam Traditional Territory

2405 Wesbrook Mall | Vancouver, BC  Canada V6T 1Z3

Phone: 604-822-7898 | Fax: 604-822-3035

[fawziah.lalji@ubc.ca](mailto:fawziah.lalji@ubc.ca) | [@ubcpharmacy](https://twitter.com/ubcpharmacy)

[www.pharmsci.ubc.ca](https://pharmsci.ubc.ca/home) | [www.facebook.com/ubcpharmacy](http://www.facebook.com/ubcpharmacy)
