# Supplementary material for: Incidence of invasive pneumococcal disease after introduction of the 13-valent conjugate pneumococcal vaccine in British Columbia: A retrospective cohort study
Source: PLoS One. 2020 Sep 30;15(9):e0239848. doi: 10.1371/journal.pone.0239848 (PMC7526878; doi:10.1371/journal.pone.0239848)
Supplement: Attachment — Submitted filename: Response to Reviewers_v5.doc [file pone.0239848.s003.doc]

September 3, 2020

Ka Chun Chong

Academic Editor

PLOS ONE

Dear Dr Chong

**RE: Manuscript Number: PONE-D-20-08116**

Thank you for re-reviewing our paper entitled “Incidence of invasive pneumococcal disease after introduction of the 13-valent conjugate pneumococcal vaccine in British Columbia: a retrospective cohort study” for consideration of publication in the journal of PLOS ONE. We do appreciate the feedback received from the reviewers. As requested, I have revised the manuscript as suggested by reviewer #1, presented as track changes throughout the revised manuscript. Please see my responses to the comments below.

***Reviewer #1:***

This is a revised version of the manuscript “Incidence of invasive pneumococcal disease after introduction of the 13-valent conjugate pneumococcal vaccine in British Columbia: a retrospective color study ” by Vadlamudi et al. Authors revised parts of the manuscript according to some of the requests, and the manuscript appears quite improved, being the analysis of results more exhaustive.

Some minor remarks:

1. Line 177-178: For clarity, authors should move the sentence in lines 177-178 to line 172, before the last sentence of the first paragraph of Results.

**Done, and I’ve revised wording slightly to accommodate the move of that sentence to line 172.**

2. Authors should correct the fields in Table 2.

**I am unclear on which fields need correcting. Can you please be specific?**

3. Line 233 (new line 235): serotypes 15A, 15C, 23A, 23B, and 35F are not included in PPV23, therefore they should not be included in this paragraph.

**Yes, you are correct and I have deleted reference to serotypes 15A, 15C, 23A, 23B, and 35F.**

4. Line 349 (new line 351): word “negated” seems to be too much imperative.

**“Changed to affected”.**

***Reviewer #2:***

No revisions requested.

I look forward to hearing back from you shortly.

Sincerely,

Fawziah

**Fawziah Lalji, B.Sc.(Pharm), ACPR, Pharm.D., FCSHP**

Professor | Tong Louie Chair

Faculty of Pharmaceutical Sciences | The University of British Columbia

Vancouver Campus | Musqueam Traditional Territory

2405 Wesbrook Mall | Vancouver, BC  Canada V6T 1Z3

Phone: 604-822-7898 | Fax: 604-822-3035

[fawziah.lalji@ubc.ca](mailto:fawziah.lalji@ubc.ca) | [@ubcpharmacy](https://twitter.com/ubcpharmacy)

[www.pharmsci.ubc.ca](https://pharmsci.ubc.ca/home) | [www.facebook.com/ubcpharmacy](http://www.facebook.com/ubcpharmacy)
